# Patient autonomy in inpatient medical rehabilitation in Germany—study protocol of a multicenter cross-sectional study

**DOI:** 10.1186/s12910-025-01340-z

**Published:** 2025-12-10

**Authors:** Malte Klemmt, Dagmar Holmer, Tanja Henking, Silke Neuderth

**Affiliations:** 1 Institute for General Practice and Palliative Care, Hannover Medical School, Carl-Neuberg-Straße 1, Hannover, 30625 Germany; 2 Institute for Applied Social Sciences, Technical University of Applied Sciences Würzburg-Schweinfurt, Münzstraße 12, Würzburg, 97070 Germany

**Keywords:** Patient autonomy, Self-determination, Medical rehabilitation, Inpatient rehabilitation, Cross-sectional study, Germany

## Abstract

**Background:**

The bioethical principle of respect for patients' autonomy should be implemented in every area of health care. The German rehabilitation system has some characteristics (e.g. the prevalence of inpatient rehabilitation) that make the topic particularly relevant. The aim of the study is to determine the current state of respecting patient autonomy in inpatient medical rehabilitation in Germany and to identify barriers and promoting factors from the perspective of relevant groups.

**Methods:**

A multi-center, prospective, cross-sectional study is being conducted, including interviews with patients (*n* = 24), interviews with professionals (*n* = 21) and a survey of medical directors (*n* = 900). The empirical findings are then reviewed in three validation workshops. In addition, consensus recommendations for practice are derived by means of a Delphi process with experts (*n* = 30).

**Discussion:**

This study will address an important gap in the empirical literature by identifying the current state and needs of patients, professionals and medical directors of rehabilitation clinics regarding the practice of respecting patient autonomy in inpatient medical rehabilitation in Germany. It is expected that the results can contribute to coming closer to the ideal of respect for patient autonomy in rehabilitation practice.

**Trial registration:**

The study was registered with the German Clinical Trials Registry (ID: DRKS00035893).

## Background

Medical rehabilitation is an important part of healthcare at various stages of life if there is a risk of or already existing health-related impairment to social and occupational participation [1]. In Germany, medical rehabilitation mainly takes place on an inpatient basis, where patients usually spend a period of at least 3 weeks. Also, rehabilitation clinics in Germany are highly specialized and focus on specific diagnostic groups. Medical fields in which inpatient medical rehabilitation most frequently take place are (in order of frequency): orthopedics, psychosomatics/psychotherapy, oncology, cardiology, neurology, and internal medicine [2]. Due to the availability of specialized clinics, the rehabilitation stay is often outside of the patients´ hometown [3]. In 2023, there were over 1.000 rehabilitation clinics with more than 161.000 beds in Germany. The size of the clinics varies from 30–40 beds up to clinics with over 500 beds. The average patient occupancy rate in 2023 was 81.5% [4]. In the rehabilitation clinics, interdisciplinary teams encompass various professionals (e.g. rehabilitation physicians, physiotherapists, occupational therapists, nutritionists, psychologists) with a strong medical dominance [3]. In Germany, inpatient medical rehabilitation is financed either by the German Pension Insurance, the statutory health insurance or an accident insurance [5]. For people of working age (usually 18–67 years old), inpatient medical rehabilitation is typically financed by the German Pension Insurance. This is the case for up to two thirds of all rehabilitation procedures [6]. Almost 1.5 million rehabilitation applications were submitted to the German pension insurance in 2022 [2].

Respect for the autonomy of patients is one of the guiding bioethical principles [7]. Structures, institutions and professionals in all areas of health care are obliged to respect the autonomy of patients. The research project „[blinded for peer review]“ focuses on the autonomy of patients in inpatient medical rehabilitation in Germany.

In Sec. 1 of the German Social Code IX, self-determination is declared as a primary goal and a relevant outcome of all rehabilitation measures in Germany. In its framework, the German Pension Insurance also names self-determination and participation as overarching goals of rehabilitation [8]. Preserving and promoting the autonomy of patients in rehabilitation can be an important element in achieving these goals [9]. The principle of respecting autonomy is particularly relevant for patients in medical rehabilitation, as measures during the rehabilitation stay can intervene deeply in the physical and psycho-emotional sphere of those affected. Institutional aspects can also be significant, as medical rehabilitation in Germany is predominantly carried out on an inpatient basis [2]. Chronic illnesses, which are often treated in inpatient medical rehabilitation, are often associated with autonomy-related impairments, which makes respect for patient autonomy even more relevant [10]. The concept of patient orientation on which medical rehabilitation is based also emphasizes the importance of respect for patient autonomy [11, 12].

Senin and Meyer [9] distinguish between two levels of patient self-determination in medical rehabilitation: self-determination as an outcome or goal of rehabilitation and self-determination within the rehabilitation measures. This study primarily examines the latter level. A scoping review by Klemmt et al. [13] identified numerous potential aspects, that can promote or endanger patient autonomy in rehabilitation, in the international research literature. These aspects concern both the personal level (patients, professionals, third parties) and the structural levels of rehabilitation clinics and of the health care system. For example, aspects that endanger autonomy have been reported regarding standardization and treatment goals [14], therapeutic measures [15], inadequate qualification and sensitization of professionals [16] or regarding misconceptions of the patients themselves [17]. Autonomy-promoting aspects were identified, for example, in relation to an autonomy-adapted institutional culture [18], supporting patient involvement (e.g. through shared decision making) [19] or the characteristics of the relationship between patients and professionals [20]. There is a lack of empirical evidence regarding autonomy-related rehabilitation practice in the context of the German rehabilitation system. The autonomy-related objectives of medical rehabilitation can benefit from the knowledge gained in this study, as empirical findings regarding patient autonomy in the German setting of inpatient medical rehabilitation are generated for the first time in this study.

The aim of the study is to determine the current state of respecting patient autonomy in inpatient medical rehabilitation practice in Germany and to identify barriers and promoting factors from the perspective of relevant groups. Based on the empirical findings and normative-theoretical considerations, consensual recommendations for the practice of respecting the autonomy of patients will be derived with the involvement of experts.

The research questions are:

1. What is the current state regarding the implementation of respect for patient autonomy in rehabilitation practice? Are there diagnosis-specific differences?
2. How do the groups involved perceive patient autonomy?
3. To what extent are there difficulties in implementing the principle of respect for autonomy?
4. Can best-practice approaches for respecting patient autonomy be identified?

## Methods

### Study design

**Fig. 1:**
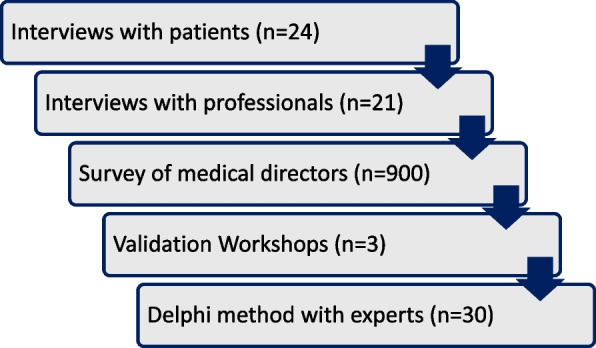
Study design

A multi-center, prospective, cross-sectional study is being conducted. As different accesses and data types are required to answer the research questions, a multi-method study design is chosen. Firstly, 1) interviews will be conducted with patients to examine the subjective experiences and attitudes of the target group. In addition, 2) interviews will be conducted with professionals to determine the perspectives of the relevant professional groups. This is followed by 3) a written survey of medical directors to investigate clinic-related and organizational characteristics. Subsequently, 4) validation workshops will be held to validate the empirical results from study parts 1–3 in a participatory manner. Finally, 5) the derived practical recommendations are consented in a Delphi process with experts and practitioners (see Fig. 1). The study was registered with the German Clinical Trials Registry (ID: DRKS00035893).

#### Interviews with patients

##### Sampling and recruitment

The recruitment of patients is carried out via eight cooperating rehabilitation clinics in different regions in Germany. The initial approach of the patients is carried out by employees of the cooperating rehabilitation clinics according to predefined inclusion and exclusion criteria. Patients who want to take part in the study and meet the inclusion criteria are then contacted by members of the project team. Inclusion criteria are: written consent to participate in the study, completion of a rehabilitation stay for a maximum of four weeks, rehabilitation in one of the most common diagnostic groups (orthopedics, psychosomatics/psychotherapy, oncology, cardiology, neurology, internal medicine [2]). Exclusion criteria are: insufficient knowledge of the German language, inability to give consent and being underaged. The aim is to contrast the sample regarding the following characteristics: age, gender and diagnostic groups. The aim is to interview four patients for each of the six most common diagnostic groups mentioned above (*n* = 24). If data saturation is not likely after the targeted interviews, further interviews will take place.

The aim is to achieve a sample size of n = 24 interviews (four cases per diagnostic group mentioned above).

##### Data collection

Data is collected by means of narrative individual interviews [21], either by telephone or video conference, depending on the participants' preference. A semi-structured interview guide and a short questionnaire on socio-demographic and health-related characteristics are used. The interviews will be conducted by a member of the project team who has been trained in conducting interviews in advance. Each interview is expected to last 45–60 min. The topics of the interviews are the subjective experience of autonomy during rehabilitation as well as perceived risks of endangerment. The interviews will be audio-recorded and transcribed verbatim.

##### Data analysis

The anonymized transcripts are analyzed using reconstructive hermeneutic text analysis [22]. The analysis is carried out by a member of the project team using MaxQDA software. (Interim) results are discussed by the entire project team. Socio-demographic information is aggregated and analyzed descriptive-statistical.

#### Interviews with professionals

##### Sampling and recruitment

The professionals are also recruited via the eight cooperating rehabilitation clinics. The initial approach is made via the medical directors according to predefined inclusion and exclusion criteria. Professionals who want to take part in the study and meet the inclusion criteria are then contacted by members of the project team. Inclusion criteria are: employment in a cooperating rehabilitation clinics, members of a profession who may come into contact with autonomy-associated constellations in the course of their work (physicians, physiotherapists and exercise therapists, nutritional therapists, occupational therapists, psychologists, social workers and nurses), working in one of the most common main diagnostic groups (orthopedics, psychosomatics/psychotherapy, oncology, cardiology, neurology, and internal medicine [2]), At least 1 year of professional experience in the current position. Exclusion criteria are: insufficient knowledge of the German language. The aim is to contrast the sample regarding the following characteristics: age, gender, professional experience. The aim is to interview three professionals for each of the six most common diagnostic groups mentioned above (*n* = 21). If data saturation is not likely after the targeted interviews, further interviews will take place.

##### Data collection

Data is collected by means of problem-centered individual interviews [23], either by telephone or video conference, depending on the participants' preference. A semi-structured interview guide and a short questionnaire on socio-demographic and job-related characteristics are used. The interviews will be conducted by a member of the project team who has been trained in conducting interviews in advance. Each interview is expected to last 45–60 min. The topics of the interviews are perceptions of patient and professional autonomy. The interviews will be audio-recorded and transcribed verbatim.

##### Data analysis

The anonymized transcripts are analyzed using content analysis according to the content-structuring qualitative content analysis method [24]. The analysis is carried out by a member of the project team using MaxQDA software. (Interim) results are discussed by the entire project team. Socio-demographic information is aggregated and analyzed descriptive-statistical.

#### Survey of medical directors

##### Sampling and recruitment

Included are all inpatient rehabilitation clinics of all diagnostic groups, treating persons of legal age in Germany that are operated by the German Pension Insurance (n = 900 [25]). Rehabilitation clinics dealing with the diagnostic groups of addiction and rehabilitation of mentally ill people are excluded. The medical directors are contacted by email via the German Pension Insurance and are invited to participate in the study. For this purpose, links/QR codes to the survey are sent in these emails.

##### Data collection

The survey is conducted using a self-constructed questionnaire based on the findings of 1) and 2). This is programmed in the SoSciSurvey program and can be answered online by the respondents. The estimated completion time is 15–20 min. Topics include the current status of respect for patient autonomy and wishes and needs from the perspective of the rehabilitation clinics.

##### Data analysis

The aggregated data from the survey is first checked for correctness and plausibility. The analysis is initially carried out descriptively by calculating frequencies and positional measures. Free text responses are categorized. Group comparisons are calculated using t-tests or chi2-tests, Eta-square coefficients or the correlation coefficient according to Bravais-Pearson, depending on the scale level of the variables examined. The analysis is carried out using SPSS.

#### Validation workshops

##### Sampling and recruitment

Participants in the validation workshops are interviewees (patients (*n *= 5) and professionals (*n* = 5)) as well as surveyed medical directors (*n* = 5). The sample size depends on an adequate workshop size and a minimum number of cases per target group. Participation in the validation workshops was approached during the data collection process (study parts 1–3). Interested persons can register here to take part in the workshops and will be contacted by members of the project team.

##### Data collection

The validation workshops take place via video conference and are moderated by members of the project team. One workshop is held for each survey group (patients, professionals, medical directors). In the workshops, results will be presented by the project team and discussed with the participants. The expected duration is 90 min each. Data will be collected using standardized discussion and results protocols.

##### Data analysis

The protocols of the validation workshops are analyzed according to the content-structuring qualitative content analysis [24]. The results serve as a validation grid for the empirical results from study parts 1–3.

#### Delphi method

##### Sampling and recruitment

Participants are recruited via a research advisory board accompanying the project as well as via other channels and personal contact. Scientific experts from relevant disciplines (medicine, health services research, social sciences, psychology, law, ethics), practitioners (professionals, management functions), patient representatives and stakeholders (including insurance companies) will be included. All potential participants will be contacted separately by email and invited to participate. A total of *n* = 30 people will take part in the Delphi process, because this number has been shown to be appropriate in previous studies.

##### Data collection

The empirically derived recommendations are consented in a two- to three-stage Delphi process [26]. For this purpose, the recommendations developed by the project team are placed in the SoSciSurvey program and can be evaluated by the participants regarding content and formal aspects using rating scales and free text fields for alternative proposals. Following the first Delphi round, the recommendations are revised and then presented to the participants again. The aim is to achieve a consensus rate of min = 80% per recommendation. If this is not achieved in the second Delphi round, a further Delphi round will take place.

##### Data analysis

The participants' assessments (rating scales) are evaluated descriptively. The free text responses are categorized and reviewed for content.

## Discussion

This study will address an important gap in the current empirical literature by identifying the current state and needs of patients, professionals and medical directors of rehabilitation clinics regarding the practice of respecting patient autonomy in inpatient medical rehabilitation in Germany. Based on the empirical findings and normative-theoretical considerations, consensual recommendations for the practice of respecting the autonomy of patients will be derived with the involvement of experts. We thus expect that the study output will be of interest to a wide range of knowledge users including scientists, stakeholders, professionals, patient groups and clinic representatives. We also expect that the results can contribute to coming closer to the ideal of respect for patient autonomy in rehabilitation practice, e.g. by identifying barriers and promoting factors.

Expected results could concern organizational/institutional, structural as well as personal or relationship aspects. We suspect that there may be a gap between self-determination as an objective or outcome of rehabilitation and respect for patient autonomy during the inpatient stay. We also suspect that there may be differences in terms of diagnostic groups (e.g. neurology vs. orthopedics).

The strengths of the study are that the relevant groups and perspectives are included. In addition, the surveys are multi-center and cross-indication, which means that bias potential can be avoided. The multi-method approach means that the research questions can be adequately addressed in each case. Challenges could relate to the response to the medical directors' survey. In addition, before starting the empirical surveys, a theoretical-normative classification of the theoretical construction of patient autonomy in medical rehabilitation must be carried out to be able to clearly define the topic of the study. This is done through extensive literature research.

## Data Availability

No datasets were generated or analysed during the current study.
